# 5-Methyl­phenanthro[2,3-*b*]thio­phene

**DOI:** 10.1107/S1600536811014838

**Published:** 2011-04-29

**Authors:** S. Ranjith, A. SubbiahPandi, V. Dhayalan, A. K. MohanaKrishnan

**Affiliations:** a Department of Physics, Presidency College (Autonomous), Chennai 600 005, India; b Department of Organic Chemistry, University of Madras, Guindy Campus, Chennai 600 025, India

## Abstract

The title compound, C_17_H_12_S, which crystallises with two molecules in the asymmetric unit, features four fused rings forming an essentially planar mol­ecule, with maximum deviations from the mean plane of 0.078 (2) and 0.080 (2) Å for C atoms of the thio­phene and phenanthrene groups in both the mol­ecules. The crystal packing features weak C—H⋯π inter­actions.

## Related literature

For a related structure, see: Gunasekaran *et al.* (2010 ▶).
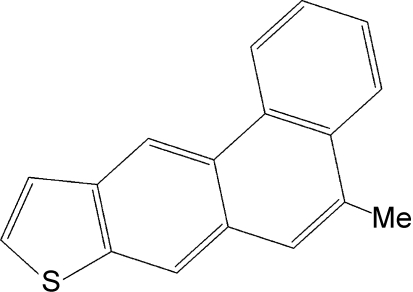


## Experimental

### 

#### Crystal data

C_17_H_12_S
*M* *_r_* = 248.33
Monoclinic, 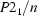

*a* = 18.7011 (10) Å
*b* = 5.8199 (3) Å
*c* = 23.4546 (14) Å
β = 105.158 (2)°
*V* = 2463.9 (2) Å^3^
*Z* = 8
Mo *K*α radiation
μ = 0.24 mm^−1^
*T* = 293 K
0.25 × 0.22 × 0.19 mm

#### Data collection

Bruker APEXII CCD area-detector diffractometer
Absorption correction: multi-scan (*SADABS*; Sheldrick, 1996 ▶) *T*_min_ = 0.981, *T*_max_ = 0.985
31917 measured reflections
6983 independent reflections
4446 reflections with *I* > 2σ(*I*)
*R*_int_ = 0.032

#### Refinement

*R*[*F* ^2^ > 2σ(*F* ^2^)] = 0.055
*wR*(*F* ^2^) = 0.195
*S* = 1.06
6983 reflections
327 parameters
2 restraints
H-atom parameters constrained
Δρ_max_ = 0.47 e Å^−3^
Δρ_min_ = −0.30 e Å^−3^

### 

Data collection: *APEX2* (Bruker, 2004 ▶); cell refinement: *APEX2*; data reduction: *SAINT* (Bruker, 2004 ▶); program(s) used to solve structure: *SHELXS97* (Sheldrick, 2008 ▶); program(s) used to refine structure: *SHELXL97* (Sheldrick, 2008 ▶); molecular graphics: *ORTEP-3* (Farrugia, 1997 ▶); software used to prepare material for publication: *SHELXL97* and *PLATON* (Spek, 2009 ▶).

## Supplementary Material

Crystal structure: contains datablocks global, I. DOI: 10.1107/S1600536811014838/bt5506sup1.cif

Structure factors: contains datablocks I. DOI: 10.1107/S1600536811014838/bt5506Isup2.hkl

Supplementary material file. DOI: 10.1107/S1600536811014838/bt5506Isup3.cml

Additional supplementary materials:  crystallographic information; 3D view; checkCIF report

## Figures and Tables

**Table 1:** Hydrogen-bond geometry (Å, °)

*D*—H⋯*A*	*D*—H	H⋯*A*	*D*⋯*A*	*D*—H⋯*A*
C2—H2⋯*Cg*4^i^	0.93	2.84	3.612 (3)	141
C2′—H2′⋯*Cg*14^ii^	0.93	2.85	3.619 (3)	141
C11—H11⋯*Cg*1^i^	0.93	2.72	3.515 (3)	144
C11′—H11′⋯*Cg*11^ii^	0.93	2.82	3.610 (3)	143
C17′—H17*B*⋯*Cg*14^iii^	0.96	2.98	3.581 (3)	121
C17—H17*F*⋯*Cg*4^iii^	0.96	2.92	3.565 (3)	125

Symmetry codes: (i) 
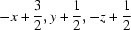
; (ii) 
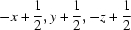
; (iii) 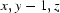. *Cg*1, *Cg*4, *Cg*11 and *Cg*14 are the centroids of the S1/C1–C4, C9–C14, S1′/C1′–C4′ and C9′–C14′ rings, respectively.

**Fig. 1.**
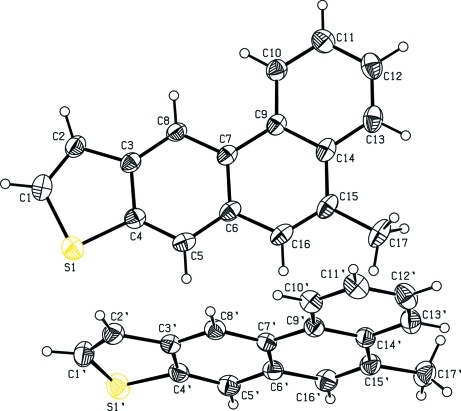
The structure of showing the atom-numbering scheme and intramolecular hydrogen bond. Displacement ellipsoids are drawn at the 30% probability level.

**Fig. 2.**
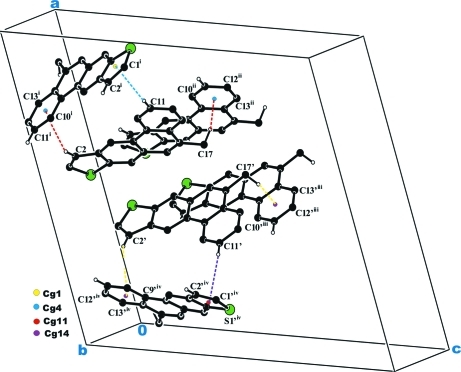
C—H···π interactions (dotted lines) in the title compound. Cg denotes ring centeroid. [Symmetry code:(i) 3/2-X,1/2+Y,1/2-Z; (ii) X,-1+Y,Z; (iii) X,-1+Y,Z; (iv) 1/2-X,1/2+Y,1/2-Z]

## supplementary crystallographic information

### Comment

X-Ray analysis confirms the molecular structure and atom connectivity as illustrated in Fig. 1. The bond lengths and angles in (Fig. 1) agree with those observed in other benzothiophene derivative (Gunasekaran *et al.*, 2010). Both   molecules are essentially planar, with maximum deviation of 0.078 (2) and 0.080 (2)Å for atoms C2 and C10'.     The molecules lack hydrogen bonding functionality and pack in layers parallel to the (010) planes.In addition to van der Waals interaction, the crystal packing is stabilized by C–H···π hydrogen bonds (Table. 1).

### Experimental

To a solution of diethyl-2-((3-(bromomethyl)thiophen-2-yl)methylene) malonate (1 g, 2.88 mmol) in dry 1,2-dichloroethane  (10ml), 1-methylnaphthalene (0.49 g, 3.44 mmol) and anhydrous ZnBr_2_ (0.64 g, 2.84 mmol) were added. sIt was then stirred at room temperature for 8 h and then refluxed for 1 h under N_2_ atmosphere. The solvent was removed and the residue was quenched with ice-water (50 ml) containing 1 ml of conc. HCl, extracted with chloroform (3 *x* 10 ml) and dried (Na_2_SO_4_). Removal of the solvent followed by flash column chromatography purification (n-hexane/ethyl acetate 99:1) led to the isolation of 5-methyl phenanthro[2,3-*b*]thiophene as a colorless crystals. Single crystals suitable for X-ray diffraction were obtained by slow evaporation of a solution of the title compound in methanol at room temperature.

### Refinement

All H atoms were fixed geometrically and allowed to ride on their parent C atoms, with C—H distances fixed in the range 0.93–0.97 Å with *U*_iso_(H) = 1.5*U*_eq_(C) for methyl H 1.2*U*_eq_(C) for other H atoms. The displacement ellipsoids of C1' and C2' were restrained to be equal within an effective e.s.d. of 0.005.

### Crystal data

**Table:**

C_17_H_12_S	*F*(000) = 1040
*M**_r_* = 248.33	*D*_x_ = 1.339 Mg m^−^^3^
Monoclinic, *P*2_1_/*n*	Mo *K*α radiation, λ = 0.71073 Å
Hall symbol: -P 2yn	Cell parameters from 6983 reflections
*a* = 18.7011 (10) Å	θ = 1.3–29.7°
*b* = 5.8199 (3) Å	µ = 0.24 mm^−^^1^
*c* = 23.4546 (14) Å	*T* = 293 K
β = 105.158 (2)°	Block, colourless
*V* = 2463.9 (2) Å^3^	0.25 × 0.22 × 0.19 mm
*Z* = 8	

### Data collection

**Table:**

Bruker APEXII CCD area-detector diffractometer	6983 independent reflections
Radiation source: fine-focus sealed tube	4446 reflections with *I* > 2σ(*I*)
graphite	*R*_int_ = 0.032
ω and φ scans	θ_max_ = 29.7°, θ_min_ = 1.3°
Absorption correction: multi-scan (*SADABS*; Sheldrick, 1996)	*h* = −25→26
*T*_min_ = 0.981, *T*_max_ = 0.985	*k* = −8→8
31917 measured reflections	*l* = −32→32

### Refinement

**Table:**

Refinement on *F*^2^	Primary atom site location: structure-invariant direct methods
Least-squares matrix: full	Secondary atom site location: difference Fourier map
*R*[*F*^2^ > 2σ(*F*^2^)] = 0.055	Hydrogen site location: inferred from neighbouring sites
*wR*(*F*^2^) = 0.195	H-atom parameters constrained
*S* = 1.06	*w* = 1/[σ^2^(*F*_o_^2^) + (0.1022*P*)^2^ + 0.5162*P*] where *P* = (*F*_o_^2^ + 2*F*_c_^2^)/3
6983 reflections	(Δ/σ)_max_ = 0.001
327 parameters	Δρ_max_ = 0.47 e Å^−^^3^
2 restraints	Δρ_min_ = −0.30 e Å^−^^3^

### Special details

**Table:**

Geometry. All e.s.d.'s (except the e.s.d. in the dihedral angle between two l.s. planes) are estimated using the full covariance matrix. The cell e.s.d.'s are taken into account individually in the estimation of e.s.d.'s in distances, angles and torsion angles; correlations between e.s.d.'s in cell parameters are only used when they are defined by crystal symmetry. An approximate (isotropic) treatment of cell e.s.d.'s is used for estimating e.s.d.'s involving l.s. planes.
Refinement. Refinement of *F*^2^ against ALL reflections. The weighted *R*-factor *wR* and goodness of fit *S* are based on *F*^2^, conventional *R*-factors *R* are based on *F*, with *F* set to zero for negative *F*^2^. The threshold expression of *F*^2^ > σ(*F*^2^) is used only for calculating *R*-factors(gt) *etc*. and is not relevant to the choice of reflections for refinement. *R*-factors based on *F*^2^ are statistically about twice as large as those based on *F*, and *R*- factors based on ALL data will be even larger.

### Fractional atomic coordinates and isotropic or equivalent isotropic displacement parameters (Å^2^)

**Table:**

	*x*	*y*	*z*	*U*_iso_*/*U*_eq_	
C1'	0.33522 (15)	0.2874 (5)	0.07093 (11)	0.0702 (7)	
H1'	0.3227	0.3551	0.0337	0.084*	
C1	0.55737 (13)	0.7887 (4)	0.06335 (10)	0.0601 (6)	
H1	0.5490	0.8546	0.0261	0.072*	
C2	0.60214 (13)	0.8818 (4)	0.11144 (10)	0.0516 (5)	
H2	0.6274	1.0193	0.1114	0.062*	
C2'	0.31625 (12)	0.3776 (4)	0.11600 (10)	0.0538 (5)	
H2'	0.2897	0.5136	0.1144	0.065*	
C3	0.60700 (11)	0.7451 (3)	0.16387 (9)	0.0431 (4)	
C3'	0.34245 (11)	0.2359 (3)	0.16957 (9)	0.0449 (4)	
C4	0.56138 (11)	0.5470 (3)	0.15084 (9)	0.0457 (4)	
C4'	0.38181 (11)	0.0412 (3)	0.15862 (9)	0.0451 (4)	
C5	0.55612 (11)	0.3950 (3)	0.19459 (10)	0.0487 (5)	
H5	0.5255	0.2669	0.1856	0.058*	
C5'	0.41350 (11)	−0.1131 (4)	0.20223 (10)	0.0496 (5)	
H5'	0.4394	−0.2397	0.1939	0.059*	
C6'	0.40615 (10)	−0.0763 (3)	0.25924 (9)	0.0438 (4)	
C6	0.59727 (11)	0.4349 (3)	0.25262 (9)	0.0446 (4)	
C7	0.64532 (10)	0.6288 (3)	0.26671 (9)	0.0411 (4)	
C7'	0.36605 (10)	0.1153 (3)	0.27204 (8)	0.0406 (4)	
C8'	0.33449 (11)	0.2692 (3)	0.22664 (9)	0.0461 (4)	
H8'	0.3079	0.3951	0.2345	0.055*	
C8	0.64871 (11)	0.7820 (3)	0.22128 (9)	0.0437 (4)	
H8	0.6794	0.9101	0.2298	0.052*	
C9'	0.35973 (10)	0.1459 (3)	0.33238 (9)	0.0431 (4)	
C9	0.68848 (11)	0.6597 (3)	0.32765 (8)	0.0416 (4)	
C10'	0.31838 (11)	0.3236 (4)	0.34787 (10)	0.0517 (5)	
H10'	0.2927	0.4236	0.3187	0.062*	
C10	0.74001 (12)	0.8395 (4)	0.34412 (9)	0.0489 (5)	
H10	0.7475	0.9395	0.3153	0.059*	
C11'	0.31458 (12)	0.3548 (5)	0.40558 (11)	0.0601 (6)	
H11'	0.2870	0.4754	0.4149	0.072*	
C11	0.77956 (13)	0.8707 (4)	0.40190 (10)	0.0567 (5)	
H11	0.8128	0.9921	0.4119	0.068*	
C12'	0.35180 (13)	0.2064 (5)	0.44909 (10)	0.0620 (6)	
H12'	0.3492	0.2269	0.4878	0.074*	
C12	0.76975 (13)	0.7205 (4)	0.44519 (10)	0.0597 (6)	
H12	0.7965	0.7414	0.4843	0.072*	
C13'	0.39265 (13)	0.0289 (4)	0.43557 (10)	0.0573 (6)	
H13'	0.4176	−0.0697	0.4653	0.069*	
C13	0.72101 (13)	0.5424 (4)	0.43057 (10)	0.0561 (6)	
H13	0.7153	0.4424	0.4600	0.067*	
C14'	0.39736 (10)	−0.0063 (3)	0.37744 (9)	0.0444 (4)	
C14	0.67910 (12)	0.5061 (3)	0.37200 (9)	0.0459 (5)	
C15'	0.44026 (11)	−0.1964 (4)	0.36283 (10)	0.0502 (5)	
C15	0.62704 (12)	0.3163 (4)	0.35639 (10)	0.0503 (5)	
C16	0.59013 (12)	0.2845 (4)	0.29960 (10)	0.0516 (5)	
H16	0.5584	0.1591	0.2901	0.062*	
C16'	0.44238 (12)	−0.2279 (4)	0.30675 (10)	0.0517 (5)	
H16'	0.4684	−0.3534	0.2980	0.062*	
C17'	0.48297 (13)	−0.3532 (4)	0.41088 (11)	0.0644 (6)	
H17A	0.5035	−0.4779	0.3935	0.097*	
H17B	0.4503	−0.4131	0.4328	0.097*	
H17C	0.5223	−0.2686	0.4369	0.097*	
C17	0.61402 (14)	0.1585 (4)	0.40359 (12)	0.0658 (7)	
H17D	0.5801	0.0392	0.3856	0.099*	
H17E	0.5935	0.2445	0.4304	0.099*	
H17F	0.6602	0.0911	0.4248	0.099*	
S1	0.51607 (4)	0.53496 (12)	0.07611 (3)	0.0620 (2)	
S1'	0.38490 (4)	0.03471 (13)	0.08505 (3)	0.0655 (2)	

### Atomic displacement parameters (Å^2^)

**Table:**

	*U*^11^	*U*^22^	*U*^33^	*U*^12^	*U*^13^	*U*^23^
C1'	0.0742 (16)	0.0787 (17)	0.0525 (13)	−0.0132 (14)	0.0075 (12)	0.0174 (12)
C1	0.0656 (14)	0.0640 (14)	0.0512 (12)	0.0067 (11)	0.0162 (10)	0.0135 (11)
C2	0.0656 (13)	0.0447 (11)	0.0494 (12)	−0.0016 (10)	0.0235 (10)	0.0059 (9)
C2'	0.0551 (12)	0.0461 (11)	0.0569 (13)	0.0003 (9)	0.0087 (10)	0.0039 (10)
C3	0.0492 (10)	0.0382 (9)	0.0471 (10)	0.0007 (8)	0.0218 (8)	0.0017 (8)
C3'	0.0431 (10)	0.0442 (10)	0.0451 (10)	−0.0050 (8)	0.0073 (8)	−0.0005 (8)
C4	0.0462 (10)	0.0438 (10)	0.0495 (11)	−0.0004 (8)	0.0171 (9)	−0.0004 (9)
C4'	0.0434 (10)	0.0489 (11)	0.0426 (10)	−0.0073 (8)	0.0103 (8)	−0.0040 (9)
C5	0.0506 (11)	0.0390 (10)	0.0594 (13)	−0.0073 (8)	0.0193 (10)	−0.0007 (9)
C5'	0.0480 (11)	0.0456 (11)	0.0548 (12)	0.0030 (9)	0.0130 (9)	−0.0045 (9)
C6'	0.0407 (10)	0.0410 (10)	0.0475 (11)	0.0008 (8)	0.0077 (8)	−0.0011 (8)
C6	0.0484 (10)	0.0378 (10)	0.0541 (12)	0.0000 (8)	0.0248 (9)	0.0038 (8)
C7	0.0473 (10)	0.0356 (9)	0.0466 (10)	0.0028 (8)	0.0232 (8)	0.0019 (8)
C7'	0.0368 (9)	0.0419 (10)	0.0416 (10)	−0.0029 (7)	0.0075 (7)	−0.0029 (8)
C8'	0.0467 (10)	0.0422 (10)	0.0483 (11)	0.0028 (8)	0.0105 (8)	−0.0032 (9)
C8	0.0555 (11)	0.0358 (9)	0.0447 (10)	−0.0065 (8)	0.0219 (9)	−0.0012 (8)
C9'	0.0379 (9)	0.0459 (10)	0.0449 (10)	−0.0017 (8)	0.0098 (8)	−0.0009 (8)
C9	0.0482 (10)	0.0391 (10)	0.0434 (10)	0.0057 (8)	0.0225 (8)	0.0021 (8)
C10'	0.0453 (10)	0.0554 (12)	0.0540 (12)	0.0048 (9)	0.0124 (9)	−0.0041 (10)
C10	0.0553 (11)	0.0482 (11)	0.0473 (11)	−0.0019 (9)	0.0208 (9)	0.0000 (9)
C11'	0.0498 (12)	0.0714 (15)	0.0624 (14)	0.0064 (11)	0.0205 (10)	−0.0104 (12)
C11	0.0562 (12)	0.0620 (13)	0.0537 (13)	0.0018 (11)	0.0178 (10)	−0.0061 (11)
C12'	0.0543 (12)	0.0867 (18)	0.0479 (12)	−0.0021 (12)	0.0187 (10)	−0.0068 (12)
C12	0.0649 (14)	0.0696 (15)	0.0446 (12)	0.0140 (12)	0.0145 (10)	−0.0021 (11)
C13'	0.0513 (12)	0.0740 (15)	0.0457 (12)	−0.0044 (11)	0.0113 (9)	0.0064 (11)
C13	0.0670 (14)	0.0600 (13)	0.0466 (12)	0.0194 (11)	0.0244 (10)	0.0099 (10)
C14'	0.0374 (9)	0.0502 (11)	0.0436 (10)	−0.0045 (8)	0.0072 (8)	0.0029 (9)
C14	0.0542 (11)	0.0428 (10)	0.0483 (11)	0.0135 (8)	0.0272 (9)	0.0082 (8)
C15'	0.0454 (10)	0.0468 (11)	0.0546 (12)	−0.0005 (9)	0.0060 (9)	0.0073 (9)
C15	0.0589 (12)	0.0428 (11)	0.0599 (13)	0.0107 (9)	0.0346 (10)	0.0149 (9)
C16	0.0565 (12)	0.0402 (10)	0.0648 (14)	−0.0026 (9)	0.0280 (10)	0.0101 (9)
C16'	0.0501 (11)	0.0443 (11)	0.0591 (13)	0.0091 (9)	0.0113 (9)	0.0001 (10)
C17'	0.0607 (14)	0.0607 (14)	0.0667 (15)	0.0089 (11)	0.0077 (11)	0.0168 (12)
C17	0.0743 (15)	0.0596 (14)	0.0740 (16)	0.0110 (12)	0.0380 (13)	0.0299 (12)
S1	0.0603 (4)	0.0672 (4)	0.0536 (4)	−0.0073 (3)	0.0061 (3)	0.0019 (3)
S1'	0.0684 (4)	0.0788 (4)	0.0530 (4)	−0.0042 (3)	0.0223 (3)	−0.0040 (3)

### Geometric parameters (Å, °)

**Table:**

C1'—C2'	1.310 (3)	C9'—C14'	1.417 (3)
C1'—S1'	1.725 (3)	C9—C10	1.407 (3)
C1'—H1'	0.9300	C9—C14	1.417 (3)
C1—C2	1.331 (3)	C10'—C11'	1.386 (3)
C1—S1	1.729 (3)	C10'—H10'	0.9300
C1—H1	0.9300	C10—C11	1.376 (3)
C2—C3	1.447 (3)	C10—H10	0.9300
C2—H2	0.9300	C11'—C12'	1.379 (3)
C2'—C3'	1.476 (3)	C11'—H11'	0.9300
C2'—H2'	0.9300	C11—C12	1.387 (3)
C3—C8	1.385 (3)	C11—H11	0.9300
C3—C4	1.419 (3)	C12'—C13'	1.371 (3)
C3'—C8'	1.398 (3)	C12'—H12'	0.9300
C3'—C4'	1.411 (3)	C12—C13	1.364 (4)
C4—C5	1.378 (3)	C12—H12	0.9300
C4—S1	1.737 (2)	C13'—C14'	1.404 (3)
C4'—C5'	1.373 (3)	C13'—H13'	0.9300
C4'—S1'	1.742 (2)	C13—C14	1.408 (3)
C5—C6	1.397 (3)	C13—H13	0.9300
C5—H5	0.9300	C14'—C15'	1.459 (3)
C5'—C6'	1.396 (3)	C14—C15	1.455 (3)
C5'—H5'	0.9300	C15'—C16'	1.339 (3)
C6'—C7'	1.419 (3)	C15'—C17'	1.507 (3)
C6'—C16'	1.444 (3)	C15—C16	1.343 (3)
C6—C7	1.426 (3)	C15—C17	1.507 (3)
C6—C16	1.441 (3)	C16—H16	0.9300
C7—C8	1.404 (3)	C16'—H16'	0.9300
C7—C9	1.457 (3)	C17'—H17A	0.9600
C7'—C8'	1.399 (3)	C17'—H17B	0.9600
C7'—C9'	1.461 (3)	C17'—H17C	0.9600
C8'—H8'	0.9300	C17—H17D	0.9600
C8—H8	0.9300	C17—H17E	0.9600
C9'—C10'	1.395 (3)	C17—H17F	0.9600
			
C2'—C1'—S1'	115.65 (19)	C11'—C10'—H10'	119.2
C2'—C1'—H1'	122.2	C9'—C10'—H10'	119.2
S1'—C1'—H1'	122.2	C11—C10—C9	121.5 (2)
C2—C1—S1	114.30 (17)	C11—C10—H10	119.3
C2—C1—H1	122.9	C9—C10—H10	119.3
S1—C1—H1	122.9	C12'—C11'—C10'	119.7 (2)
C1—C2—C3	112.4 (2)	C12'—C11'—H11'	120.1
C1—C2—H2	123.8	C10'—C11'—H11'	120.1
C3—C2—H2	123.8	C10—C11—C12	119.9 (2)
C1'—C2'—C3'	111.3 (2)	C10—C11—H11	120.1
C1'—C2'—H2'	124.3	C12—C11—H11	120.1
C3'—C2'—H2'	124.3	C13'—C12'—C11'	120.3 (2)
C8—C3—C4	119.30 (18)	C13'—C12'—H12'	119.8
C8—C3—C2	129.41 (19)	C11'—C12'—H12'	119.8
C4—C3—C2	111.29 (19)	C13—C12—C11	120.2 (2)
C8'—C3'—C4'	118.48 (18)	C13—C12—H12	119.9
C8'—C3'—C2'	130.0 (2)	C11—C12—H12	119.9
C4'—C3'—C2'	111.52 (19)	C12'—C13'—C14'	121.0 (2)
C5—C4—C3	121.2 (2)	C12'—C13'—H13'	119.5
C5—C4—S1	127.90 (17)	C14'—C13'—H13'	119.5
C3—C4—S1	110.93 (15)	C12—C13—C14	121.6 (2)
C5'—C4'—C3'	122.2 (2)	C12—C13—H13	119.2
C5'—C4'—S1'	127.17 (17)	C14—C13—H13	119.2
C3'—C4'—S1'	110.59 (15)	C13'—C14'—C9'	119.2 (2)
C4—C5—C6	119.36 (19)	C13'—C14'—C15'	121.09 (19)
C4—C5—H5	120.3	C9'—C14'—C15'	119.68 (19)
C6—C5—H5	120.3	C13—C14—C9	118.5 (2)
C4'—C5'—C6'	118.82 (19)	C13—C14—C15	121.65 (19)
C4'—C5'—H5'	120.6	C9—C14—C15	119.8 (2)
C6'—C5'—H5'	120.6	C16'—C15'—C14'	119.61 (19)
C5'—C6'—C7'	120.86 (18)	C16'—C15'—C17'	120.4 (2)
C5'—C6'—C16'	120.13 (18)	C14'—C15'—C17'	120.0 (2)
C7'—C6'—C16'	118.95 (18)	C16—C15—C14	119.40 (18)
C5—C6—C7	120.71 (18)	C16—C15—C17	120.3 (2)
C5—C6—C16	120.42 (19)	C14—C15—C17	120.3 (2)
C7—C6—C16	118.84 (19)	C15—C16—C6	123.3 (2)
C8—C7—C6	118.51 (19)	C15—C16—H16	118.4
C8—C7—C9	122.88 (18)	C6—C16—H16	118.4
C6—C7—C9	118.61 (17)	C15'—C16'—C6'	123.1 (2)
C8'—C7'—C6'	118.90 (18)	C15'—C16'—H16'	118.5
C8'—C7'—C9'	122.30 (18)	C6'—C16'—H16'	118.5
C6'—C7'—C9'	118.80 (17)	C15'—C17'—H17A	109.5
C3'—C8'—C7'	120.70 (19)	C15'—C17'—H17B	109.5
C3'—C8'—H8'	119.7	H17A—C17'—H17B	109.5
C7'—C8'—H8'	119.7	C15'—C17'—H17C	109.5
C3—C8—C7	120.92 (18)	H17A—C17'—H17C	109.5
C3—C8—H8	119.5	H17B—C17'—H17C	109.5
C7—C8—H8	119.5	C15—C17—H17D	109.5
C10'—C9'—C14'	118.04 (19)	C15—C17—H17E	109.5
C10'—C9'—C7'	122.22 (18)	H17D—C17—H17E	109.5
C14'—C9'—C7'	119.73 (18)	C15—C17—H17F	109.5
C10—C9—C14	118.38 (19)	H17D—C17—H17F	109.5
C10—C9—C7	121.69 (17)	H17E—C17—H17F	109.5
C14—C9—C7	119.93 (18)	C1—S1—C4	91.03 (11)
C11'—C10'—C9'	121.7 (2)	C1'—S1'—C4'	90.93 (12)
			
S1—C1—C2—C3	1.1 (3)	C6—C7—C9—C14	−3.5 (3)
S1'—C1'—C2'—C3'	−0.4 (3)	C14'—C9'—C10'—C11'	1.0 (3)
C1—C2—C3—C8	178.3 (2)	C7'—C9'—C10'—C11'	−178.1 (2)
C1—C2—C3—C4	−1.1 (3)	C14—C9—C10—C11	−1.4 (3)
C1'—C2'—C3'—C8'	179.8 (2)	C7—C9—C10—C11	178.70 (19)
C1'—C2'—C3'—C4'	0.6 (3)	C9'—C10'—C11'—C12'	−0.6 (4)
C8—C3—C4—C5	2.0 (3)	C9—C10—C11—C12	1.0 (3)
C2—C3—C4—C5	−178.43 (19)	C10'—C11'—C12'—C13'	0.2 (4)
C8—C3—C4—S1	−178.83 (15)	C10—C11—C12—C13	0.0 (3)
C2—C3—C4—S1	0.7 (2)	C11'—C12'—C13'—C14'	−0.2 (4)
C8'—C3'—C4'—C5'	−1.2 (3)	C11—C12—C13—C14	−0.5 (3)
C2'—C3'—C4'—C5'	178.05 (19)	C12'—C13'—C14'—C9'	0.6 (3)
C8'—C3'—C4'—S1'	−179.91 (15)	C12'—C13'—C14'—C15'	−179.4 (2)
C2'—C3'—C4'—S1'	−0.6 (2)	C10'—C9'—C14'—C13'	−0.9 (3)
C3—C4—C5—C6	−0.9 (3)	C7'—C9'—C14'—C13'	178.15 (18)
S1—C4—C5—C6	−179.89 (16)	C10'—C9'—C14'—C15'	179.06 (18)
C3'—C4'—C5'—C6'	0.3 (3)	C7'—C9'—C14'—C15'	−1.9 (3)
S1'—C4'—C5'—C6'	178.75 (16)	C12—C13—C14—C9	0.1 (3)
C4'—C5'—C6'—C7'	0.7 (3)	C12—C13—C14—C15	−179.93 (19)
C4'—C5'—C6'—C16'	−176.45 (19)	C10—C9—C14—C13	0.9 (3)
C4—C5—C6—C7	−1.0 (3)	C7—C9—C14—C13	−179.26 (17)
C4—C5—C6—C16	177.11 (19)	C10—C9—C14—C15	−179.12 (17)
C5—C6—C7—C8	1.8 (3)	C7—C9—C14—C15	0.8 (3)
C16—C6—C7—C8	−176.34 (18)	C13'—C14'—C15'—C16'	178.6 (2)
C5—C6—C7—C9	−178.46 (17)	C9'—C14'—C15'—C16'	−1.4 (3)
C16—C6—C7—C9	3.4 (3)	C13'—C14'—C15'—C17'	−2.5 (3)
C5'—C6'—C7'—C8'	−0.7 (3)	C9'—C14'—C15'—C17'	177.52 (19)
C16'—C6'—C7'—C8'	176.44 (18)	C13—C14—C15—C16	−177.8 (2)
C5'—C6'—C7'—C9'	179.81 (18)	C9—C14—C15—C16	2.2 (3)
C16'—C6'—C7'—C9'	−3.0 (3)	C13—C14—C15—C17	2.9 (3)
C4'—C3'—C8'—C7'	1.2 (3)	C9—C14—C15—C17	−177.15 (18)
C2'—C3'—C8'—C7'	−177.96 (19)	C14—C15—C16—C6	−2.3 (3)
C6'—C7'—C8'—C3'	−0.2 (3)	C17—C15—C16—C6	177.00 (19)
C9'—C7'—C8'—C3'	179.21 (17)	C5—C6—C16—C15	−178.7 (2)
C4—C3—C8—C7	−1.2 (3)	C7—C6—C16—C15	−0.5 (3)
C2—C3—C8—C7	179.37 (19)	C14'—C15'—C16'—C6'	2.4 (3)
C6—C7—C8—C3	−0.7 (3)	C17'—C15'—C16'—C6'	−176.44 (19)
C9—C7—C8—C3	179.61 (17)	C5'—C6'—C16'—C15'	177.0 (2)
C8'—C7'—C9'—C10'	3.6 (3)	C7'—C6'—C16'—C15'	−0.2 (3)
C6'—C7'—C9'—C10'	−176.94 (18)	C2—C1—S1—C4	−0.56 (19)
C8'—C7'—C9'—C14'	−175.43 (18)	C5—C4—S1—C1	178.9 (2)
C6'—C7'—C9'—C14'	4.0 (3)	C3—C4—S1—C1	−0.11 (16)
C8—C7—C9—C10	−3.9 (3)	C2'—C1'—S1'—C4'	0.0 (2)
C6—C7—C9—C10	176.38 (17)	C5'—C4'—S1'—C1'	−178.2 (2)
C8—C7—C9—C14	176.23 (17)	C3'—C4'—S1'—C1'	0.36 (16)

### Hydrogen-bond geometry (Å, °)

**Table:**

*D*—H···*A*	*D*—H	H···*A*	*D*···*A*	*D*—H···*A*
C2—H2···Cg4^i^	0.93	2.84	3.612 (3)	141
C2'—H2'···Cg14^ii^	0.93	2.85	3.619 (3)	141
C11—H11···Cg1^i^	0.93	2.72	3.515 (3)	144
C11'—H11'···Cg11^ii^	0.93	2.82	3.610 (3)	143
C17'—H17B···Cg14^iii^	0.96	2.98	3.581 (3)	121
C17—H17F···Cg4^iii^	0.96	2.92	3.565 (3)	125

Symmetry codes: (i) −*x*+3/2, *y*+1/2, −*z*+1/2; (ii) −*x*+1/2, *y*+1/2, −*z*+1/2; (iii) *x*, *y*−1, *z*.
